# Inefficiencies in a healthcare system with a regulatory split of power: a spatial panel data analysis of avoidable hospitalisations in Austria

**DOI:** 10.1007/s10198-019-01113-7

**Published:** 2019-09-09

**Authors:** Anna-Theresa Renner

**Affiliations:** Health Economics and Policy Group, Vienna University of Economics and Business (WU), Welthandelsplatz 1, Building D4, 1020 Vienna, Austria

**Keywords:** Avoidable hospitalisations, Inefficiencies in healthcare, Outpatient service quality, Cost containment, Health systems, I11, I18, H51

## Abstract

Despite generous universal social health insurance with little formal restrictions of outpatient utilisation, Austria exhibits high rates of avoidable hospitalisations, which indicate the inefficient provision of primary healthcare and might be a consequence of the strict regulatory split between the Austrian inpatient and outpatient sector. This paper exploits the considerable regional variations in acute and chronic avoidable hospitalisations in Austria to investigate whether those inefficiencies in primary care are rather related to regional healthcare supply or to population characteristics. To explicitly account for inter-regional dependencies, spatial panel data methods are applied to a comprehensive administrative dataset of all hospitalisations from 2008 to 2013 in the 117 Austrian districts. The initial selection of relevant covariates is based on Bayesian model averaging. The results of the analysis show that supply-side variables, such as the number of general practitioners, are significantly associated with decreased chronic and acute avoidable hospitalisations, whereas characteristics of the regional population, such as the share of population with university education or long-term unemployed, are less relevant. Furthermore, the spatial error term indicates that there are significant spatial dependencies between unobserved characteristics, such as practice style or patients’ utilization behaviour. Not accounting for those would result in omitted variable bias.

## Introduction

Healthcare expenditures represent a substantial part of the public budget in many European countries^1^ and are projected to increase due to demographic changes and increasingly expensive new treatments. Based on these projections and in light of the most recent economic crises, reducing or limiting the growth of healthcare spending are considered relevant austerity measures [2]. One possibility to achieve such reductions in publicly financed healthcare systems is to decrease hospital costs. Without jeopardizing the health of the population, this goal can best be attained by increasing efficiency and accessibility of primary healthcare^2^ and therefore reducing avoidable hospitalisations [4].

Given that healthcare can be received free of economically relevant co-payments, a sick person’s decision if, where and when to enter the healthcare system depends (1) on the probability that the health problem will dissolve (i.e. subjective need), (2) on the individual (time) preferences of the patient, and (3) on the relative disutility of visiting a doctor. This relative disutility is determined by the availability (i.e. geographic reachability) and the accessibility (i.e. opening hours and waiting times) of the primary healthcare provider compared to alternatives (e.g. the hospital). Hence, environmental factors, such as the distribution of physicians, are an important determinant of healthcare utilisation. Austria is a country with almost no formal restrictions to healthcare utilization and also exhibits one of the highest rates of avoidable hospitalisations for diabetes, coronary heart failure and COPD in Europe [5]. This is despite the fact that Austria has the highest physician density per population in the European Union. Of the total 5.13 practising physicians per 1000 population in 2016, 2.34 were practising in the outpatient sector [6]. The rates of avoidable hospitalisations are not only comparably high, the differences between Austrian regions are also substantial with a range of 22–56 per 1000 population. The same is true for the outpatient physician density per 1000 population, ranging from 0.14 in Rust to almost 65 in the city of Graz [6]. However, whether a service is actually available and accessible for a particular patient depends on the patient’s individual characteristics such as age, gender, disability and socioeconomic status (which can also influence individual (time) preferences) [7]. The aim of this paper, therefore, is to examine whether the regional differences in avoidable hospitalisations are due to inadequate distribution of outpatient physician supply or to demand-side characteristics. In particular, the analysis focuses on the socioeconomic variations across the country as a source of demand-side predictor of avoidable hospitalizations, under the assumption that under a well-functioning health system providing universal health coverage, social and economic factors should play no role in defining such outcomes.

The drivers of regional variations in avoidable hospitalisation rates as a measure for timely and effective primary care have been investigated before. Several studies have found a significantly negative association between avoidable hospitalisations and socioeconomic factors such as education and income using cross-sectional data [8–12]. The impact of the quantity of certain ambulatory health services on avoidable hospitalisation was studied by Sundmacher and Kopetsch [13], who used administrative data form the ambulatory sector in Germany. They found that additional monetary resources for ambulatory services reduce the hospitalisation rates for these conditions [13]. Similar results were found for Switzerland and France [14, 15]. A study conducted in the USA confirmed the effect of primary care practitioners on reducing avoidable hospitalisations for urban but not for rural areas [16]. A recent study by Whittaker et al. [17] compared the emergency department visits of patients registered at primary care practices with extended opening hours (evenings and weekends) to patients registered with other primary care practices in the Greater Manchester area. They found a 26% relative reduction for emergency care visits for minor health problems only within the first year [17]. For Austria, Czypionka et al. could not confirm these results. They did not find a significant relationship between avoidable hospitalisation rates with neither socioeconomic characteristics, nor outpatient service provision [18]. However, they did find a significant association between the amount of services provided in outpatient acute day wards and same-day hospital treatments (admissions without overnight stay) with avoidable hospitalisation [18].

All of the existing studies use cross-sectional study designs (with the exception of Berlin et al. [14] who include year dummies) and most of them do not account for possible inter-regional spillovers (except for [13, 15]). With a comprehensive dataset that captures all avoidable hospitalisations in Austria from 2008 to 2013, I am able to apply spatial panel data methods that allow to control for time invariant regional heterogeneity while at the same time accounting for spatial dependencies between districts.

The paper proceeds as follows: in the first part, the particularities of the Austrian healthcare system with its complex financing structures and the regulatory split between outpatient and inpatient sector is presented. In the second part, the empirical methods for the spatial panel data analysis are described and its results presented. Finally, the discussion brings together the institutional and empirical parts of the paper.

## Institutional background

Austria poses an interesting example of a country with universal social health insurance covering an extensive set of services with almost no formal restrictions of utilisation for the patient, but with a rather strict divide of regulative power between hospital and outpatient sector. In a recent report on the financing of the Austrian public healthcare system, the Austrian Court of Audit heavily criticized the fragmentation of competences regarding regulation, provision and especially the allocation of funds. It elaborates that despite efforts, notably during a health system reform in 2013, responsibility and accountability for expenditures, functioning and financing of the healthcare system are still split up among the social insurance funds, the federal state and the *Bundesländer* (provinces).

To provide a clear overview of this complex system, a classification framework proposed by [19] will be applied to the Austrian inpatient (i.e. hospitals including ambulatory care wards) and outpatient healthcare sector (i.e. independent physicians providing primary or secondary care). It considers regulation, provision and financing as three dimensions of healthcare systems.

### Regulation

By constitution, the Austrian Federal Government is responsible for the overall health policy which includes the monitoring of the health insurance funds as well as the law-making and its implementation. However, the nine provinces are in charge of laws of execution and their implementation within the hospital sector [20]. Furthermore, the constitutional responsibility for the social security system, which includes not only health but also accident and pension insurance, has been delegated from the federal state level to the self-governing insurance funds. Health insurance is mandatory and every Austrian resident is automatically assigned to one of the funds depending on her occupational status and place of residence. Additionally, around 35% (2013) of the population holds supplementary health insurance from a private insurer. Private health insurance policies mainly cover additional amenities in the hospital, such as single or twin rooms, as well as private physicians in the outpatient sector. The General Social Insurance Act (*Allgemeines Sozialversicherungsgesetz, ASVG*) sets out the general rules and frame for the Austrian social security system. In particular, it regulates the contributions and coverage of the pension, accident and health insurance funds and the relationship between the funds and other stakeholders. The detailed organisation and financing of the healthcare system are based on federal law and several binding agreements between the federal state and the provinces. Based on these documents, the “*Österreichischer Strukturplan Gesundheit”* (Austrian structural plan for health; *ÖSG*) is issued by an expert advisory panel and forms the basis for the (hospital) service planning [21].

In addition, a placement plan (*Stellenplan*) for public (i.e. contracted) physicians in each region is negotiated between each social health insurance fund and the regional chambers of physicians (*Landes¨arztekammern*). By law, the distribution of these contracted physicians has to account for regional differences in infrastructure such that every insured person can choose between at least two different contracted physicians reachable in due time. The location of private physicians, on the other hand, is unregulated. The strict regulation of contracted physicians is related to the high overall number of practising physicians in Austria which again is a consequence of the unregulated access to medical universities until 2005. In other countries, such as Germany or Switzerland, the contracting of physicians is only restricted if over-provision of a certain specialisation is observed in a region [22].

The general tariffs for the services are negotiated between the different health insurance funds and the chamber of physicians and apply to all contracted physicians. This means that selective contracting for certain services of physicians with the insurances is not possible, as it is in other health insurance countries such as Germany, France or Switzerland [22]. The provision and financing of public inpatient services, on the other hand, is based on a binding agreement between the federal state and the nine provinces. The agreement is renegotiated every 5–10 years and has to be passed by parliament [23].

### Provision

In 2016, 273 hospitals were operating in Austria of which 117 were general hospitals (according to ICHA-HP Classification of health care providers by the OECD) Of these general hospitals 81 were publicly owned (i.e. by provinces or municipalities), 24 were private for-profit and 11 were private non-profit institutions. All of them operate under public law and are, therefore, required to provide care to everybody in need [24]. Despite efforts to strengthen the primary care sector, the hospital sector is still dominating the Austrian healthcare system. This is reflected by the high rate of hospital discharges and acute care hospital beds per 1000 inhabitants (256 and 5.84, respectively), which exceed the European Union averages 173 and 3.94 per 1000 population. Only Germany and Bulgaria provide more acute care beds, and only Bulgaria records a higher rate of hospital discharges. This is despite the fact that the average length of stay in Austrian hospitals is 6.5 days which is almost the same as the EU average of 6.4 [24].

Outpatient services are mainly provided by physicians practising independently in single or, to a much lesser extent, in group practices. They can be classified into contracted and non-contracted physicians, the former holding a contract with at least one social health insurance fund. In 2016 a total of 23,091 outpatient general practitioners, specialists and dentists, practised, of which around 45% held a public insurance contract. The lowest share of contracted physicians can be found among the specialists (31%), the highest among general practitioners (56%) (data source: Gesundheit Österreich GmbH).

Patients are free to choose any outpatient physician, and only have to pay out-of-pocket if the chosen physician does not hold a contract with the patient’s insurance fund. A small share of this out-of-pocket payment is reimbursed by the patient’s insurance fund if medical necessity is acknowledged. Patients are therefore not restricted by regional boarders, but might be by financial restraints if they choose to see an uncontracted doctor.

### Financing

Austria’s health expenditures (29,454.9 Mio in 2009, 33,316.6 in 2013 and 36,876 Mio in 2016) are mainly financed by public funds which are subdivided into government (referring to taxes collected at national and subnational level) and social health insurance schemes (i.e. compulsory contributions). During the last decade, the former covered around 30% of total expenditures on health and the latter around 44%. Voluntary health insurance schemes and private out-of-pocket payments accounted for up to 26% (source: OECD statistics, 2018). The share of financing through government funds is rather high compared to other social insurance countries in Europe. In 2013, France, Germany and Switzerland funded only 4, 7 and 19%, respectively, of their total health expenditures through government funds. Compared to the OECD34 average, Austria exhibited higher shares of health expenditures funded through social security contributions in 2013 (45% verses 36%) [1].

The contributions for the social security funds are deducted directly from the income of the insured. However, the pooling and allocation mechanisms differ between inpatient and outpatient sector. The expenses of the Austrian acute hospitals are reimbursed based on a DRG-like (diagnosis-related groups) system (the so-called *Leistungsorientierte Krankenanstaltenfinanzierung, LKF*) through the health funds of the nine provinces (*Landesgesundheitsfonds*) and to a smaller extent directly by the provinces and municipalities. The *Landesgesundheitsfonds* receive their resources from (1) the social insurance funds (a lump sum of around 4.8 million € in 2014), (2) tax revenues from the provinces and municipalities (over 4 million € in 2014), and (3) other sources and funds including general and value-added taxes from the federal state (around 1 million €) (for further details see [23, 24]). Despite the distribution of funds to the hospitals based on the diagnosis of the admitted patients, the global budget for each province is fixed prospectively. This means that there is no direct financial incentive for hospitals to over provide as the value of each DRG point depends on the total amount of points within a province. However, it might incentivise shifting patients to outpatient care or to another province [20].

The non-hospital ambulatory services, mainly provided by independent physicians, are reimbursed on a fee-for-service basis with additional contact-based payments (i.e. for the first contact with a patient within one quarter). Physicians that hold a contract with one of the 18 health insurance funds receive payments from the social health insurance funds according to the respective tariff catalogue (*Tarifkatalog*). Non-contracted physicians can set their fees freely and are paid by the patient out-of-pocket, who can then request reimbursement of up to 80% of the official fee in the tariff catalogue. Around 18% of all expenditure for medical practices that provide ambulatory care was financed by household out-of-pocket payments, and 71% by health insurance schemes (source: OECD statistics, 2018).

It has repeatedly been pointed out that the split of power between the outpatient and inpatient sector concerning regulation, planning and financing, impedes reforms and policies that aim to decrease inefficiencies at the interface of both sectors [22, 24–26]. Avoidable hospitalisations are one of these inefficiencies, and, as described above, can be related to the supply (distribution) of services and to demand-side characteristics of the population. In the remainder of this paper, regional differences in avoidable hospitalisations will be analysed regarding their supply- and demand-side drivers.

## Empirical analysis

In 1992, Weissman et al. [27] proposed an extensive list of so-called “Ambulatory Care Sensitive Conditions”^3^ (ACSC) as a measure for accessible and effective primary healthcare. ACSCs are acute and chronic clinical indications,^4^ which operationalize the concept of (potentially) avoidable hospitalizations (i.e. hospitalisation avoidable through outpatient care). They are therefore defined as hospitalizations that could potentially be avoided by prevention, treatment or disease management in the ambulatory or outpatient sector [28] (also see [4, 29] for a validation of ACSC as a measure of potentially avoidable hospitalisations). Table 1 lists the conditions, whether they are classified as chronic or acute, and the respective diagnoses (according to ICD-10 codes) that are identified as ambulatory care sensitive.

**Table 1:** Relevant ACSC and the respective ICD-10 codes and categories

Condition	Classification	ICD 10 code
Angina	Acute	I20, I24.0, I24.8, I24.9
Asthma	Chronic	J45, J46
Cardiovascular diseases	Chronic	I13.0, I25, I48
Cellulitis	Acute	I89.1, L01, L02, L03, L04, L08.0, L08.8, L08.9, L88, L98.0, L98.3
Congestive heart failure	Chronic	I11.0, I50, J81
Convulsions and epilepsy	Chronic	G40, G41, R56
COPD	Chronic	J20, J41, J42, J43, J44, J47
Dehydration and gastroenteritis	Acute	E86, K52.2, K52.8, K52.9
Diabetes	Chronic	E10.0–10.8, E11.0–11.8, E12, E13.0–13.8, E14.0–14.8, E16.2
Diseases of the blood	Chronic	D51, D52
Ear, nose, throat infections	Acute	H66, H67, J02, J03, J04, J06, J31.2
Gangrene	Acute	R02
Hypertension	Chronic	I10, I11.9
Influenza and pneumonia	Acute	J10, J11, J13, J14, J15.3, J15.4, J15.7, J15.9, J16.8, J18.1, J18.8
Iron deficiency anaemia	Chronic	D50.1, D50.8, D50.9
Nutritional deficiencies	Chronic	E40, E41, E42, E43, E55.0, E64.3
Pelvic inflammatory disease	Acute	N70, N73, N74
Perforated/bleeding ulcer	Acute	K20, K21, K25.0–25.2, K25.4–25.6, K26.0–26.2, K26.4–26.6, K27.0–27.2, K27.4–27.6, K28.0–28.2, K28.4–28.6
Urinary tract infection	Acute	N10, N11, N12, N13.6, N15.9, N30.0, N30.8, N30.9, N39.0

*ICD* international classification of diseases, *COPD* chronic obstructive pulmonary disease

**Fig. 1:**
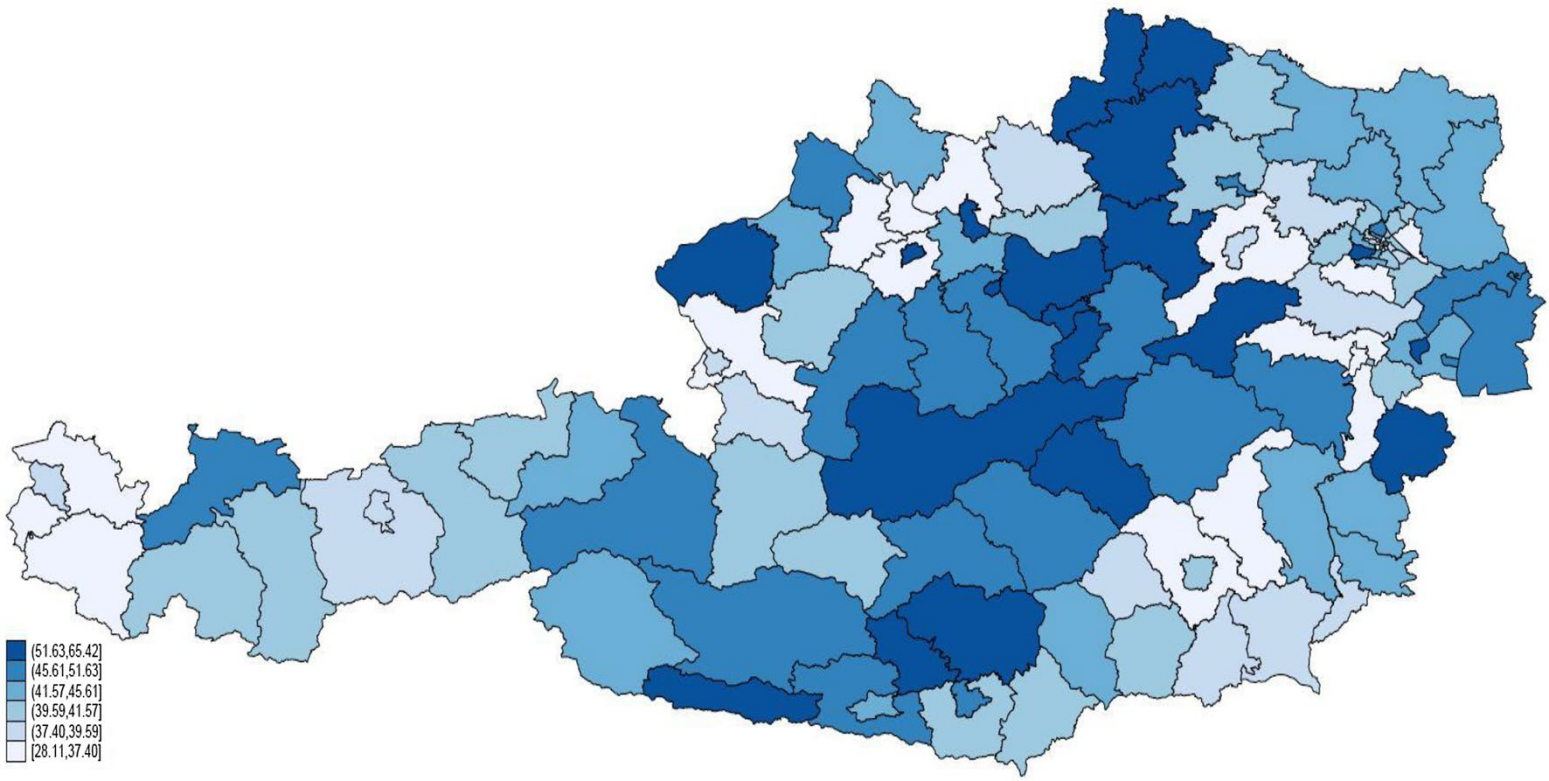
Regional distribution of the share of avoidable hospitalisations in 2013 (in quintiles)

In 2013, avoidable hospitalisations accounted for 705,584,399 LKF points, corresponding to almost 10% of all LKF points reported by all public hospitals in Austria. The rate of hospitalisations due to ACSC (i.e. AH) differs substantially between the 117 political districts, ranging from 22 to over 56 avoidable hospitalisations per 1000 inhabitants in 2013. The same is true for the share of AH on general hospital admissions (see Fig. 1), which on average is around 11%.

To investigate the drivers of these regional variations, a panel dataset containing information about hospitalisation rates for all Austrian political districts from 2008 to 2013 was exploited. Hospitalisations for all ACSC as a share of all general hospitalisations was used as the dependent variable (Y = avoidable hospitalisations/general hospitalisations) to control for reversed causality between the hospital utilization and some of the explanatory supply-side variables. The underlying assumption is that outpatient physicians are likely to base their location decision on the need of the regional population and that this need is better reflected by the number of general hospitalisations than by the regional population size. In accordance with previous literature [8, 9, 11, 12, 14, 15] only the main diagnosis of each hospital stay was considered. I first analyse all conditions jointly and then split them into chronic and acute conditions as outlined in Table 1. The aim of this separate analysis is to elicit whether the data generating processes behind those two is similar, or whether they need to be treated as two inherently different outcomes. The hospitalisation data, including the ICD 10 main diagnosis on the district level, were provided by the Austrian Ministry of Health (now: Ministry of Labour, Social Affairs, Health and Consumer Protection) and are available upon formal request only.

### Explanatory variables

The included explanatory variables (for descriptive statistics see Table 4 in the Appendix) are clustered around three categories: socioeconomic status (SES), healthcare supply (HCS) and demographics (DM). Socioeconomic status of the population is measured using education, employment status, social benefit recipients and net income. Healthcare supply includes a range of variables reflecting in- and outpatient healthcare supply (outpatient physician density, age and gender, hospital beds and physicians). Finally, DM includes variables, such as age, gender, population density and care allowance recipients. It is important to note that the set of diagnoses defined as “ambulatory care sensitive” are based on conditions that can be treated in an ambulatory care setting. This means that the hospitalisation rates due to those diagnoses (e.g. diabetes mellitus with renal complications) should be independent of actual prevalence rates for the underlying conditions (e.g. diabetes mellitus type 2), therefore ruling out that an association between increased ACSC rates and e.g. socioeconomic status can be explained by different levels of morbidity. However, to still account for latent variables that are time invariant, such as general morbidity and prevalence of chronic diseases, as well as general time trends, I include time and regional fixed effects in my regression analysis. This makes it possible to estimate the associations between avoidable hospitalisation rates and socioeconomic, as well as healthcare supply variables, for a given level of time invariant morbidity levels. The main explanatory variables are described below.

### Socioeconomic variables

Average educational attainment in a region cannot only inform about the opportunities of the population on the job market but has also been shown to be associated with health (system) literacy [30]. The latter is an important precondition for effective utilisation of available healthcare services and enables patients to find the best point of service and eventually prevent hospitalisation. Furthermore, demonstrated lower health literacy of socioeconomically deprived patients might increase opportunities for supply-side inducement including increased referrals to the hospital [31]. This is especially relevant for countries that do not exert any direct restrictions on the utilization of healthcare services, and which might, therefore, suffer, not only from supply-side inducement of demand but also from consumer moral hazard; both of these behaviours likely lead to substantial inefficiencies in the healthcare market [32]. Additionally, higher educational attainment decreases the risk of job loss and increase employability [33]. Persons in insecure employment situations might try to postpone time-consuming treatments as much as possible in fear of losing their job. The shares of population between 25 and 64 with compulsory schooling (ISCED^5^ 1 and 2), (post-)secondary schooling (ISCED 3–5) and university degree (ISCED 6 and higher) as highest educational attainment were obtained from the Statistics Austria’s register of educational attainment [6].

Population income can influence regional avoidable hospitalisation rates as people might have more financial means to access outpatient primary care and therefore avoid hospitalisations. Low income, might hinder the accessibility of available (outpatient) services due to lower levels of free disposable time and financial means (e.g. to pay for childcare during the visit). This might delay necessary treatment and lead to more extensive treatments or hospitalisations in advanced stages of the disease. Income data from 2008 to 2013 was obtained from the Statistics Austria’s database on income taxes [6].

In the empirical analysis, two variables reflect a district’s employment level: unemployment rate (number of people receiving unemployment benefits per population aged 15–64) and the rate of *Notstandshilfe*^6^ recipients. The unemployment rate reflects the short-term working status of a population and might have a positive effect on avoidable hospitalisations as it can facilitate access to primary care due to more free disposable time, i.e. less restrictions regarding the opening hours of physician offices. The same positive impact on healthcare utilization can be expected for the long-term unemployed who receive *Notstandshilfe*. To account for endogeneity from reversed causality, the previous year’s rates are included in the analysis. This lag implies a delayed effect of unemployment on health by a year. Data were retrieved from the Integrated Wage and Income Tax Statistics [6].

### Healthcare supply variables

The second group of variables that is of interest to explain variations in avoidable hospitalisation rates reflects the supply-side of the healthcare system. Based on the definition of AH as hospitalisations that can be avoided by effective and timely ambulatory or outpatient care, I am interested in whether the amount of outpatient physicians, has a significant effect on avoidable hospitalisation rates. Therefore, the density of outpatient general practitioners (GPs) and specialists per 1000 inhabitants are included in the empirical analysis. Outpatient specialists can be expected to affect avoidable hospitalisation rates for two reasons: (1) some specialists (e.g. for internal medicine) also provide preventive and primary care services and (2) GPs might want to refer patients to specialists (e.g. to perform certain diagnostic tests). Endogeneity might be an issue if outpatient physicians choose their place of work based on the AH rates within a given year. Such an instant reaction of the supply-side to an indirect measure of demand for primary care is not very likely. Furthermore, the panel structure of the data allows controlling for time-invariant endogeneity caused by different regional levels of unmet outpatient need.

Gender and age of outpatient physicians is included in the analysis as well. The gender of outpatient physicians can reflect their impact on service supply as female physicians tend to work part-time more often. This is especially true in rural areas, where child-care facilities are usually scarce and traditional gender roles are persistent. The age of outpatient physicians is included as a proxy for their experience, the continuity of care and the mutual trust between physician and patient. Younger outpatient physicians might be less confident and therefore more inclined to refer patients to the hospital. Unfortunately, empirical studies that investigate this assumption could not be found. Older outpatient physicians, on the other hand, might benefit from higher levels of trust associated with better adherence [34]. The data on outpatient physicians was provided by the Austrian Public Health Institute (Gesundheit Österreich GmbH).

Hospital characteristics reflecting inpatient supply are regarded in the analysis for two reasons: (1) to control for substitution of ambulatory services in (outpatient) hospital day-wards and (2) to take into account the reachability of the hospitals in case of an emergency. Two different measures of hospital resources were included: the number of hospital beds and the number of hospital physicians in full-time-equivalents. As hospital resources are planned on the basis of need, reversed causality could pose a threat to the validity of the results. However, a change in avoidable hospitalisations would only impact the planned hospital resources of the following year. Furthermore, using the general hospitalisation rates as the denominator of the dependent variable should rid these effects. Variables reflecting the level of available hospital services, such as the number of hospital beds per 1000 inhabitants and the number of hospital physicians (full-time equivalents), were obtained from the hospital statistics of the Austrian Ministry of Health.

### Estimation strategy

The structural form of the constrained regional and time fixed effects model without any spatial lags is the following:

$${\text{AH}}_{i,t} = \alpha _{i} + \tau _{t} + \beta _{{\rm SES}} {\text{SES}}_{i,t} + \beta _{{\rm HCS}} {\text{HCS}}_{i,t} + \beta _{{\rm DM}} {\text{DM}}_{i,t } + \varepsilon _{i,t} ,$$

with *t* and *i* as time and regional indexes. Demeaning the dependent and the independent variables by their regional averages eliminates the regional fixed effects *α*, and all time-invariant variables from the model. Furthermore, including time fixed effects allows to control for a possible time trend.

**Fig. 2:**
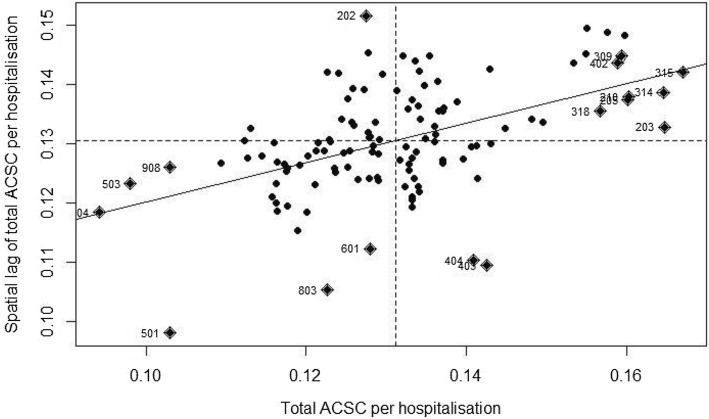
Moran’s I test of spatial autocorrelation for total avoidable hospitalisations. Data from 2013 and a queen contiguity spatial weights matrix have been used

As patients in Austria are not restricted to certain regional service suppliers when seeking outpatient care, characteristics of geographically close districts are conceivably of high importance. Furthermore, latent common factors such as practice style or ‘culture’ of utilisation might lead to spatial spillovers. The Moran’s *I* test confirms the presence of spatial autocorrelation in the share of total avoidable on general hospitalisations (*I* = 0.332 with *p* = 0.000) (see Fig. 2 in the Appendix). It is, therefore, necessary to account for spatial dependencies when estimating the independent effects of demand- and supply-side characteristics on avoidable hospitalisations in order to avoid endogeneity bias from omitted variables. The explicit modelling of spatial dependencies also allows the estimation and interpretation of global and local spatial spillovers between regions for certain variables.

To account for spatial dependencies in a fixed-effects panel data model, independent, dependent or error terms can be spatially weighted and included as right-hand-side variables. Including all three, results in a model of the following structural form: $y_{i,t } = \alpha_{i,t} + \lambda Wy_{i,t} + \beta_{x} X_{i,t} + \theta WX_{i,t} + u_{i,t} ,$ with $u_{i,t} = \rho Wu_{i,t} + \varepsilon_{i,t}$ and $\varepsilon_{i,t} \sim iid\left( {0, \;\sigma^{2} I_{i,t} } \right)$. *W* is a predefined *N* × *N* spatial weights matrix (SWM) with elements *ω*_*i,t*_ that measure the closeness of regions *i* and *j*. The value of *ω*_*i,t*_ depends on the measure of closeness deployed. Details on the creation and standardization of the SWM can be found in the Appendix [34, 35].

If both, *λ* and *θ*, from the unconstrained model are zero, the Spatial Error Model (SEM) is the true data generating process (DGP). This means that unmodelled, latent effects spillover across regions corresponding to the spatial multiplier. The SEM is also a way of modelling spatial heterogeneity, where closer regions should exhibit more similar effect levels than regions that are further away [35]. The SDEM is a local spillover model, as regions’ outcomes are affected by the explanatory variables of their own as well as their neighbouring regions. Additionally, the model accounts for spatial autocorrelation in the error terms. All mentioned spatial models can be estimated consistently and unbiased using maximum likelihood.^7^

The spatial dependencies of avoidable hospitalisations can either be attributed to spillovers in the healthcare supply between districts (e.g. because patients utilize doctors in other districts) or because of dependencies in latent variables such as similar treatment patterns of doctors or utilization behaviours of patients in closer-by districts. The former theoretical model would imply a Spatial Durbin Error Model (SDEM) with weighted healthcare supply variables, and the latter a Spatial Error Model (SEM). The SEM allows for spatially correlated omitted variables but is also consistent with global diffusion of shocks throughout the disturbance terms (i.e. global spillovers from one region to its neighbours, its neighbours’ neighbours and so on) [34]. Following this reasoning, the SEM and the SDEM were estimated and tested to elicit which model reflects the underlying data generating process.

For the initial selection of relevant covariates for the spatial panel regression, a Bayesian model sampling (BMS) was performed on linear fixed effects (FE) models. This approach was used to choose between potentially relevant but highly correlated socioeconomic (share of people with university and compulsory schooling, short-term and long-term unemployment, and per capita net-income), outpatient and inpatient healthcare supply (number of GPs and specialists per population, age shares of GPs and specialists, GPs per specialists, planned and actual number of hospital beds and hospital physicians), as well as additional demographic and morbidity variables (gender and age structure of the population, population density and share of care allowance recipients). For the BMS, first, regional fixed effects were accounted for by demeaning the data. After this, year dummies were added for estimation of two-way fixed effects models (following [36, 37]). All sampled models included year dummies, share of females and age shares of the population as fixed regressors (for details on the Bayesian model sampling see Zeugner [37]). Following the BMS, the spatial error model (SEM) and the spatial Durbin error model (SDEM) were estimated including the selected variables using fixed-effects panel data estimations. Three different spatial weights matrices (SWM) were used: a row-standardized, first-order queen contiguity (SWM_qc_), a 5-nearest-neighbours (SWM_5nb_) and a distance decay with 50 km cut-off (SWM_dd_) (see Fig. 5 in the Appendix for a graphic representation). The appropriate model specification was then chosen based on the Bayesian Information Criterion (BIC) and the likelihood ratio test.

## Results

**Fig. 4:**
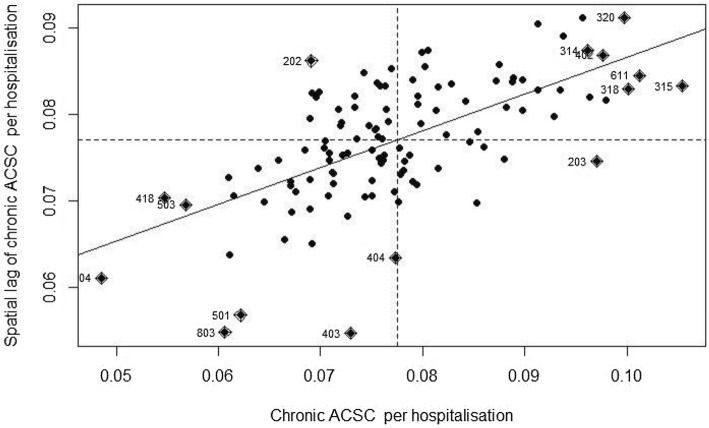
Moran’s I test of spatial autocorrelation for chronic avoidable hospitalisations. Data from 2013 and a queen contiguity spatial weights matrix have been used

**Fig. 5:**
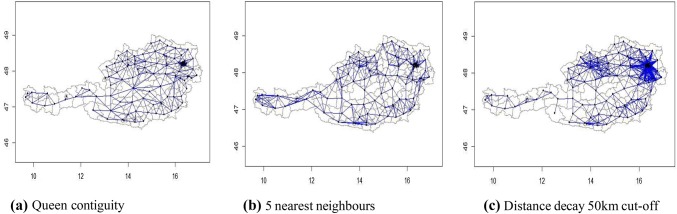
Illustration of spatial weights matrices based on three different proximity measures

Based on the BMS results (see Tables 5, 6, Fig. 4 in the Appendix) the following explanatory variables were selected for inclusion in the spatial regression models: share of population with university education, share of care allowance and *Notstandshilfe* recipients, population density, share of population aged 15–64 and aged over 70, share of female population, GPs and specialists per 1000 inhabitants, number of GPs per specialists, share of specialists over 70 and between 40 and 69 years old, and hospital physicians (full-time equivalents). A comparison of the BIC of all SEM and SDEM specifications indicates that the SEM specification is the preferred model (see Table 7 in the Appendix). This finding is confirmed by a likelihood ratio test which shows that the Null hypothesis of the additional parameters in the SDEM compared to the SEM being all zero cannot be rejected at a 99% level.

Table 2 shows the results of the SEM for avoidable hospitalisation due to total, acute and chronic conditions. The coefficient on the share of population with university education is insignificant for total, acute and chronic AH. The coefficient on the share of *Notstandshilfe* recipients on the other hand, is significantly positive for total and chronic AH, but not for acute. The share of care allowance recipients is significantly and negatively associated with total and chronic AH but insignificantly associated with acute AH.

**Table 2:** Main regression results for total, acute and chronic avoidable hospitalisations

	Total AH	Acute AH	Chronic AH
	SWM_qc_	SWM5_nb_	SWM_qc_
University education	− 0.031	0.032	− 0.050
*Notstandshilfe* recipients	0.314	− 0.293***	0.413***
Care allowance recipients	− 0.620***	− 0.169	− 0.424***
Population density	− 0.000	− 0.000	− 0.000**
Population 15 to 64	0.705***	0.120**	0.432***
Population over 65	0.733***	0.118	0.415***
Female population	0.166	− 0.148	0.286
GPs per 1000 pop.	− 0.025**	− 0.010***	− 0.015***
Specialists per 1000 pop.	0.002**	0.001	0.001
GPs per specialists	0.012***	0.006***	0.004
Specialists over 70	− 0.062***	− 0.014	− 0.046***
Specialists 40–69	− 0.019**	− 0.006	− 0.017***
Hospital physicians	− 0.000	0.000	− 0.000
Spatial error term *ρ*	0.215***	0.380***	0.199***
BIC	− 2609.06	− 3219.48	− 2911.63

All models are estimated using a Spatial Error Model with regional and time fixed effects

*AH* avoidable hospitalisations, *GP* general practitioner, *SWM* spatial weights matrix, *qc* queen contiguity, *5nb* 5-nearest neighbours, *dd* distance decay 50 km cut-off, *fte* full-time equivalent, *BIC* bayesian information criterion

***p < 0.01, **p < 0.05

There is a significantly negative association between the number of GPs per 1000 inhabitants and the share of AH in all models, whereas that between the specialist density and share of AH is insignificant for chronic conditions. The ratio between GPs and specialists on the other hand, seems to be only relevant for acute conditions but not for chronic. The opposite is the case for specialists’ age which exhibits significantly negative coefficients for chronic AH, but insignificant ones for acute AH. The number of hospital physicians in a district does not explain a significant part of the variation in total, acute and chronic AH. The estimation results consistently show significant coefficients on the population age (negative), and insignificant coefficients on the share of female population and population density.

The spatial error term *ρ* is highly significant in the SEM with queen contiguity and a 5-nearest-neighbours spatial weights matrix, but insignificant when a distance decay with a 50 km cut-off is used. Hence, not accounting for the spatial autocorrelation in the error terms would result in biased estimates. The algebraic signs as well as the magnitudes of the significant coefficients are generally robust to changes of the SWM. The BIC suggests that the queen contiguity specification should be preferred for total and chronic AH, whereas the 5-nearest neighbours SWM is more relevant for acute conditions (see Table 8 in the Appendix).

Table 3 shows the percentage point changes in each dependent variable for a typical change in selected explanatory variables, i.e. by their respective standard deviation (SD). The results show that districts with a GP density one standard deviation above the mean have a 0.97 percentage point lower share of avoidable total hospitalisations corresponding to 8.7 percent of the mean share of AH. Specialist density, on the other hand, is associated with an increased share of AH of 0.64 percentage points per standard deviation, corresponding to 5.7 percent of the mean share of AH.

**Table 3:** Percentage point change in share of total, acute and chronic AH per standard deviation of selected variables

	Total AH	Acute AH	Chronic AH
	SWM_qc_	SWM_5nb_	SWM_qc_
GPs per 1000 population	− 0.97	− 0.34	− 0.59
Specialists per 1000 population	0.64	n.s.	n.s.
GPs per specialists	0.33	0.17	n.s.
Specialists over 70 years old	− 0.11	n.s.	− 0.08
Specialists 40 to 69 years old	− 0.11	n.s.	− 0.1
Mean share of avoidable hospitalisations on overall hospitalisations (in %)	11.14	4.52	7.44

*AH* avoidable hospitalisations, *GP* general practitioner, *SWM* spatial weights matrix, *qc* queen contiguity, *5nb* 5-nearest neighbours, *n.s.* not significant at a 95%-level

## Discussion

The results of the spatial panel data regressions show that the main socioeconomic variable, university education, does not exhibit significant effects on the share of avoidable hospitalisations, whereas the share of *Notstandshilfe* recipients, representing long-term unemployment, shows highly significant coefficients in all estimated model specifications. The insignificance of education, in contrast to previous studies on avoidable hospitalisations, is likely to be due to the fixed effects regression model. Somewhat surprisingly, the share of care allowance recipients is negatively associated with avoidable hospitalisation rates. This might be due to the fact that those receiving care allowances are generally better looked after by social and healthcare providers than those who are not included in the system.

As expected, higher numbers of GPs per regional population as well as higher shares of more experienced outpatient specialists are associated with reduced avoidable hospitalisation rates. For example an increase in GPs per 1000 inhabitants by one SD is associated with a decrease in the share of total, acute and chronic AH by 8.7, 7.5 and 7.9% at the mean. The negative coefficient on GP service provision of − 0.025 on the share of total avoidable hospitalisations means that a one unit increase of GPs per 1000 population on average is associated with 590 avoidable hospitalisations less per region. This corresponds to about 1.4 million LKF points for hospital reimbursement from the public budget.^8^ Outpatient specialist density, on the other hand, is associated with increased total avoidable hospitalisations but becomes insignificant when analysing acute and chronic conditions separately. The ratio of GPs per specialists is significantly and positively associated with the share of AH for acute conditions in a region but not for chronic conditions. This might reflect referrals from GPs to hospitals in regions lacking outpatient specialist provision.

The level of hospital provision in terms of beds was found to have no, or very small, significant effects on avoidable hospitalisation rates. Hence, when accounting for general hospitalisations, the level of inpatient supply does not seem to be an inducing factor for avoidable hospitalisations. This is not to say that it might not proportionately increase both, general hospitalisations and avoidable hospitalisations, which would result in no change of the dependent variable.

The significance of the spatial error term indicates that there is variation in relevant unobserved characteristics that spillover across regions. When a distance decay with cut-off at 50 km is used as proximity measure, the spatial error term becomes zero. This could indicate that only the closest or neighbouring districts are relevant, independent of how far they are away in absolute terms. The 50 km cut-off value might lead to discarding of possibly important dependencies between remote areas where districts are larger, and over-emphasizing of dependencies in urban areas where districts are generally smaller. For conditions that lead to acute AH, the 5-nearest-neighbours SWM seems to outperform the first-order contiguity SWM. Hence, the relative distance between neighbouring districts matters more than the absolute distance.

Due to the fixed-effects model, the impact of possible determinants of avoidable hospitalisations that did not change during the study period or are homogeneous across regions, could not be estimated but are controlled for. Furthermore, the heterogeneous impact of out-of-pocket payments on the service utilization of different socioeconomic groups is captured in the socio-economic variables (i.e. unemployment rate and educational attainment). Under the applied methodology, it can, however, not be disentangled from other mechanisms through which socioeconomic status affects utilization.

The main limitation of this study is that the level of observation is the district, which might be too large to properly assess the mechanisms behind avoidable hospitalisations. This is especially relevant if the heterogeneity within a region is large and might be the reason why the socioeconomic characteristics, contrary to other studies on AH, are not significant. A second limitation is that the rate of hospitalisations for ACSC is a summary measure that aggregates selected chronic and acute conditions for which hospitalisation is necessary, but could be prevented with earlier interventions in the outpatient sector. It includes subgroups from various diagnoses that are sometimes very different regarding risk factors, aetiopathology, symptoms, and treatment. For this reason a separate analysis of acute and chronic conditions was performed, however, an in-depth analysis of single diseases (e.g. diabetes, COPD or coronary heart disease) might yield further insights into the drivers of regional variations in avoidable hospitalisations.

## Conclusions

Austria has one of the highest hospitalisation rates for diabetes, COPD and chronic heart failure, which are considered to be reducible by effective outpatient care, in Europe. At the same time, Austria is unique in its strict regulatory split between outpatient and inpatient healthcare sector. This split has been said to create or increase inefficiencies, such as avoidable hospitalisations, in the healthcare system. A necessary condition for this argument to hold is that supply-side characteristics are affecting regional rates of AH. The presented regression analysis shows that the level of outpatient healthcare provision, especially the number of GPs per population, is indeed associated with avoidable hospitalisations.

For acute conditions, the ratio of outpatient GPs and specialists is decisive, indicating that not only availability outpatient primary care but also of specialist outpatient care as a substitute of hospital care plays an important role. In order to reduce costly hospitalisations it is therefore not sufficient to ensure adequate supply of primary care physicians but also appropriate geographic distribution of outpatient specialists. However, it is important to keep in mind that there is a trade-off between spending public resources on avoidable hospitalisations in the inpatient sector and spending it on increased outpatient service provision. Furthermore, it becomes evident that hospitalisations due to acute and chronic ambulatory care sensitive conditions are driven by different characteristics of healthcare provision. While avoidable hospitalisation rates for chronic conditions are influenced by the level of experience of the available specialists, avoidable hospitalisations due to acute conditions seem to be related to their general availability. This is important for future work on ambulatory care sensitive conditions as it indicates that there are different mechanisms behind conditions categorized as chronic versus acute.

The suboptimal distribution of outpatient GPs and specialists could be counteracted by stricter placement plans for public and private outpatient physicians or by well-designed incentive schemes to steer their location choice. Attempts to foster outpatient care have been made during the 2013 healthcare reform, e.g. by establishing the legal framework for multi-disciplinary primary healthcare centres. However, to this date, only five such centres operate, two of which are located in Vienna. Future health reforms should focus on ensuring the geographic accessibility of outpatient services, especially in light of changing demographics and the increasing rural/urban divide regarding public services. Furthermore, it needs to be emphasized that, on a policy level, outpatient and inpatient services should be treated as two sides of the same coin: the public healthcare system.

## Figures and Tables

**Fig. 3:**
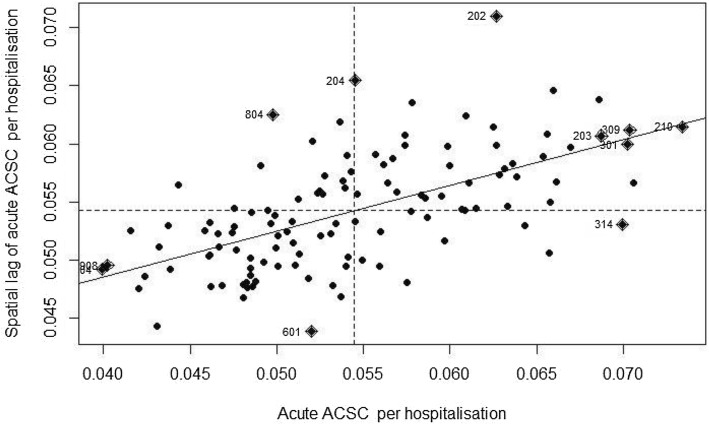
Moran’s I test of spatial autocorrelation for acute avoidable hospitalisations. Data from 2013 and a queen contiguity spatial weights matrix have been used

**Fig. 6:**
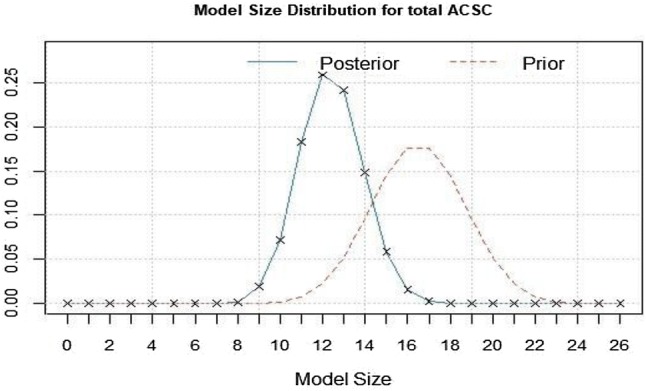
Posterior model size distribution

## Appendix

See Tables 4, 5, 6, 7, 8 and Figs. 2, 3, 4, 5, 6.

**Table 4:** Summary statistics per year

	Year	*n*	Min	Mean	Max	SD
Total ACSC per 1000 inhabitants	2009	117	23.24	38.15	65.60	6.80
	2010	117	23.75	37.37	62.67	6.67
	2011	117	22.28	36.62	55.45	6.18
	2012	117	23.08	36.65	53.62	6.48
	2013	117	22.08	36.07	56.08	6.65
	All years	585	22.08	36.97	65.60	6.58
Total ACSC per general hospitalisation	2009	117	0.08	0.12	0.17	0.01
	2010	117	0.08	0.11	0.17	0.01
	2011	117	0.08	0.11	0.15	0.01
	2012	117	0.08	0.11	0.14	0.01
	2013	117	0.07	0.11	0.15	0.01
	All years	585	0.07	0.11	0.17	0.01
Acute ACSC per general hospitalisation	2009	117	0.03	0.04	0.06	0.01
	2010	117	0.03	0.04	0.05	0.01
	2011	117	0.03	0.04	0.05	0.01
	2012	117	0.03	0.04	0.06	0.01
	2013	117	0.02	0.04	0.05	0.01
	All years	585	0.02	0.04	0.06	0.01
Chronic ACSC per general hospitalisation	2009	117	0.05	0.08	0.13	0.01
	2010	117	0.05	0.08	0.12	0.01
	2011	117	0.05	0.07	0.10	0.01
	2012	117	0.05	0.07	0.10	0.01
	2013	117	0.05	0.07	0.10	0.01
	All years	585	0.05	0.08	0.13	0.01
Unemployment rate	2009	117	0.01	0.04	0.08	0.01
	2010	117	0.01	0.04	0.08	0.01
	2011	117	0.01	0.04	0.08	0.01
	2012	117	0.01	0.04	0.09	0.02
	2013	117	0.02	0.04	0.09	0.02
	All years	585	0.01	0.04	0.09	0.01
Net income per capita	2009	117	12,415.4	14,982.95	30,032.28	2160.45
	2010	117	13,033.13	15,360.02	26,649.04	1879.8
	2011	117	13,163.91	15,913.11	27,038.65	1876.54
	2012	117	13,621.17	16,292.26	26,330.61	1821.85
	2013	117	13,589.97	16,699.32	26,392.72	1745.7
	All years	585	12,415.4	15,869.37	30,032.28	1973.61
Share of *Notstandshilfe* recipients (lagged)	2009	117	0	0.01	0.04	0.01
	2010	117	0	0.02	0.04	0.01
	2011	117	0.01	0.02	0.05	0.01
	2012	117	0	0.02	0.04	0.01
	2013	117	0.01	0.02	0.04	0.01
	All years	585	0	0.02	0.05	0.01
Share of population aged 25–64 with compulsory education	2009	117	0.11	0.18	0.31	0.05
	2010	117	0.12	0.18	0.32	0.05
	2011	117	0.11	0.18	0.32	0.05
	2012	117	0.11	0.18	0.34	0.05
	2013	117	0.11	0.18	0.35	0.05
	All years	585	0.11	0.18	0.35	0.05
Share of population aged 25–64 with university education	2009	117	0.04	0.07	0.4	0.08
	2010	117	0.04	0.07	0.41	0.09
	2011	117	0.04	0.08	0.42	0.09
	2012	117	0.04	0.08	0.43	0.09
	2013	117	0.05	0.09	0.44	0.09
	All years	585	0.04	0.08	0.44	0.09
Share of female population	2009	117	0.5	0.51	0.55	0.01
	2010	117	0.5	0.51	0.55	0.01
	2011	117	0.5	0.51	0.55	0.01
	2012	117	0.5	0.51	0.55	0.01
	2013	117	0.5	0.51	0.54	0.01
	All years	585	0.5	0.51	0.55	0.01
Share of population aged 15–64 (lagged)	2009	117	0.62	0.67	0.75	0.02
	2010	117	0.62	0.67	0.75	0.02
	2011	117	0.61	0.67	0.75	0.02
	2012	117	0.61	0.67	0.75	0.02
	2013	117	0.62	0.67	0.75	0.02
	All years	585	0.61	0.67	0.75	0.02
Share of population over 65 (lagged)	2009	117	0.13	0.17	0.25	0.02
	2010	117	0.13	0.18	0.25	0.02
	2011	117	0.13	0.18	0.25	0.02
	2012	117	0.13	0.18	0.25	0.02
	2013	117	0.13	0.18	0.25	0.02
	All years	585	0.13	0.18	0.25	0.02
Population density per residence area in km^2^	2009	117	278.13	647.28	43,465.58	8317.99
	2010	117	277.33	649.37	43,520.45	8328.1
	2011	117	276.36	645.32	43,852.12	8361.2
	2012	117	275.21	641.45	43,709.14	8385.09
	2013	117	274.1	637.44	44,116.47	8505.43
	All years	585	274.1	645.32	44,116.47	8351.14
Share of immigrated population	2009	117	0	0.01	0.07	0.01
	2010	117	0	0.01	0.08	0.01
	2011	117	0	0.01	0.08	0.01
	2012	117	0	0.01	0.09	0.01
	2013	117	0	0.01	0.1	0.02
	All years	585	0	0.01	0.1	0.01
Share of care allowance recipients (lagged)	2009	117	0.03	0.05	0.09	0.01
	2010	117	0.03	0.05	0.08	0.01
	2011	117	0.03	0.05	0.08	0.01
	2012	117	0.03	0.05	0.08	0.01
	2013	117	0.03	0.05	0.08	0.01
	All years	585	0.03	0.05	0.09	0.01
GPs per specialist	2009	117	0.14	0.59	3	0.33
	2010	117	0.12	0.58	2	0.26
	2011	117	0.13	0.59	2	0.26
	2012	117	0.14	0.57	2	0.25
	2013	117	0.13	0.56	2	0.24
	All years	585	0.12	0.58	3	0.27
GPs per 1000 inhabitants	2009	117	0.48	0.73	3.25	0.33
	2010	117	0.49	0.73	3.27	0.33
	2011	117	0.49	0.74	3.42	0.34
	2012	117	0.5	0.73	3.5	0.36
	2013	117	0.5	0.73	3.69	0.37
	All years	585	0.48	0.73	3.69	0.35
Specialists per 1000 inhabitants	2009	117	0.46	1.14	18.65	2.12
	2010	117	0.53	1.13	20.15	2.32
	2011	117	0.53	1.17	21.18	2.38
	2012	117	0.53	1.2	21.49	2.37
	2013	117	0.52	1.23	23.54	2.57
	All years	585	0.46	1.19	23.54	2.35
Share of GPs aged 40–69	2009	117	0.73	0.89	1	0.06
	2010	117	0.71	0.89	1	0.06
	2011	117	0.7	0.89	1	0.06
	2012	117	0.69	0.89	1	0.06
	2013	117	0.72	0.87	1	0.05
	All years	585	0.69	0.89	1	0.06
Share of GPs over 70	2009	117	0	0.01	0.09	0.02
	2010	117	0	0.01	0.09	0.02
	2011	117	0	0.01	0.11	0.02
	2012	117	0	0.01	0.11	0.02
	2013	117	0	0.02	0.15	0.03
	All years	585	0	0.01	0.15	0.02
Share of specialists aged 40–69	2009	117	0.71	0.91	1	0.04
	2010	117	0.74	0.91	1	0.04
	2011	117	0.64	0.9	1	0.05
	2012	117	0.65	0.9	1	0.05
	2013	117	0.62	0.89	1	0.05
	All years	585	0.62	0.9	1	0.05
Share of specialists over 70	2009	117	0	0.01	0.08	0.02
	2010	117	0	0.02	0.08	0.02
	2011	117	0	0.02	0.09	0.02
	2012	117	0	0.02	0.09	0.02
	2013	117	0	0.02	0.1	0.02
	All years	585	0	0.02	0.1	0.02
Share of male GPs	2009	117	0.32	0.66	0.94	0.13
	2010	117	0.32	0.64	0.88	0.13
	2011	117	0.31	0.63	0.87	0.13
	2012	117	0.3	0.62	0.87	0.13
	2013	117	0.31	0.61	0.92	0.13
	All years	585	0.3	0.64	0.94	0.13
Share of male specialists	2009	117	0	0.72	0.85	0.1
	2010	117	0	0.72	0.85	0.1
	2011	117	0	0.71	0.85	0.1
	2012	117	0	0.7	0.9	0.1
	2013	117	0	0.69	0.9	0.1
	All years	585	0	0.71	0.9	0.1
Hospital beds per 1000 inhabitants (actual)	2009	117	0	5.3	59.11	8.54
	2010	117	0	5.28	60.64	8.62
	2011	117	0	5.3	59.57	8.51
	2012	117	0	5.57	58.08	8.5
	2013	117	0	5.72	57.3	8.4
	All years	585	0	5.34	60.64	8.49
Hospital beds per 1000 inhabitants (planned)	2009	117	0	5.43	69.62	9.45
	2010	117	0	5.26	69.13	9.38
	2011	117	0	5.48	68.42	9.3
	2012	117	0	5.47	67.94	9.37
	2013	117	0	5.59	67.03	9.22
	All years	585	0	5.43	69.62	9.31
Number of hospital physicians (fte)	2009	117	0	67.45	1463.79	250.65
	2010	117	0	68.78	1414.35	255.7
	2011	117	0	73.53	1417.25	257.3
	2012	117	0	75.67	1429.43	257.67
	2013	117	0	64.76	1442.32	261.27
	All years	585	0	69.46	1463.79	255.67

*GP* General practitioner, *fte* full time equivalent, *n* number of observations, *Min* minimum observed value, *Max* maximum observed value, *SD* standard deviation

**Table 5:** Top 20 models from Bayesian model sampling

	M1	M2	M3	M4	M5	M6	M7	M8	M9	M10	M11	M12	M13	M14	M15	M16	M17	M18	M19	M20
Unemployment rate (lag)	0	0	0	0	0	0	0	0	0	0	0	0	0	0	0	0	0	0	0	0
Net income per capita (log)	0	0	0	0	0	0	0	0	0	0	0	0	0	0	0	0	0	0	0	0
*Notstandshilfe* recipients (lag)	0	0	0	0	0	1	0	0	0	0	0	0	0	1	0	0	0	1	0	1
Compulsory education	0	0	0	0	0	0	0	0	0	0	0	0	0	0	0	0	0	0	0	0
Univ. education	0	0	0	0	0	0	0	0	0	0	0	0	0	0	0	0	0	0	0	0
Female population	1	1	1	1	1	1	1	1	1	1	1	1	1	1	1	1	1	1	1	1
Population 15–64 (lag)	1	1	1	1	1	1	1	1	1	1	1	1	1	1	1	1	1	1	1	1
Population > 65 (lag)	1	1	1	1	1	1	1	1	1	1	1	1	1	1	1	1	1	1	1	1
Population density	0	0	0	0	0	0	1	1	0	1	1	0	0	0	1	1	0	0	0	0
Immigration	0	0	0	0	0	0	0	0	0	0	0	0	0	0	0	0	0	0	0	0
Care allowance recipients (lag)	1	1	1	1	1	1	1	1	1	1	1	1	1	1	1	1	1	1	1	1
GPs per specialists	0	1	1	0	1	1	0	1	1	0	1	0	1	0	0	1	0	1	0	0
GPs per 1000 population	1	1	1	1	1	1	1	1	1	1	1	1	1	1	1	1	1	1	1	1
Specs per 1000 population	0	1	0	1	1	1	0	0	1	0	0	0	1	0	0	0	1	1	0	1
GPs 40–69	0	0	0	0	0	0	0	0	0	0	0	0	1	0	0	0	0	0	0	0
GPs > 70	0	0	0	0	0	0	0	0	0	0	0	0	0	0	0	0	0	0	1	0
Specialists 40–69	1	1	1	1	0	1	1	0	0	0	1	0	1	1	1	1	0	0	1	1
Specialists > 70	1	1	1	1	0	1	1	0	1	0	1	0	1	1	0	0	0	0	1	1
Male GPs	0	0	0	0	0	0	0	0	0	0	0	0	0	0	0	0	0	0	0	0
Mal specialists	0	0	0	0	0	0	0	0	0	0	0	0	0	0	0	0	0	0	0	0
Hospital beds	0	0	0	0	0	0	0	0	0	0	0	0	0	0	0	0	0	0	0	0
Hospital physicians	0	0	0	0	0	0	0	0	0	0	0	0	0	0	0	0	0	0	0	0
2010	1	1	1	1	1	1	1	1	1	1	1	1	1	1	1	1	1	1	1	1
2011	1	1	1	1	1	1	1	1	1	1	1	1	1	1	1	1	1	1	1	1
2012	1	1	1	1	1	1	1	1	1	1	1	1	1	1	1	1	1	1	1	1
2013	1	1	1	1	1	1	1	1	1	1	1	1	1	1	1	1	1	1	1	1
PMP (exact)	0.05	0.04	0.03	0.02	0.02	0.01	0.01	0.01	0.01	0.01	0.01	0.01	0.01	0.01	0.01	0.01	0.01	0.01	0.01	0.01

**Table 6:** Results of BMS with time and regional fixed effects estimation

	PIP	Post Mean	Post SD	Cond.Pos.Sign
Female population	1.00	0.04	0.25	0.64
Population 15–64 (lag)	1.00	0.77	0.14	1.00
Population >65 (lag)	1.00	0.87	0.16	1.00
2010	1.00	− 0.00	0.00	0.00
2011	1.00	− 0.01	0.00	0.00
2012	1.00	− 0.01	0.00	0.00
2013	1.00	− 0.01	0.00	0.00
GPs per 1000 population	0.99	− 0.02	0.01	0.00
Care allowance recipients (lag)	0.91	− 0.54	0.24	0.00
Specialists >79	0.59	− 0.04	0.04	0.00
Specialists 40–69	0.57	− 0.01	0.01	0.00
GPs per specialist	0.53	0.01	0.01	1.00
Specialists per 1000 pop.	0.52	0.00	0.00	1.00
Population density	0.30	− 0.00	0.00	0.00
*Notstandshilfe* recipients (lag)	0.22	0.07	0.16	1.00
GPs >79	0.16	0.00	0.01	1.00
GPs 40–69	0.12	− 0.00	0.00	0.01
University education	0.08	− 0.01	0.03	0.01
Compulsory education	0.08	0.01	0.03	0.99
Male specialists	0.07	− 0.00	0.00	0.00
Net income per capita (log)	0.06	− 0.00	0.01	0.13
Unemployment rate (lag)	0.05	0.00	0.02	0.74
Male GPs	0.04	0.00	0.00	0.72
Immigration	0.04	− 0.00	0.03	0.37
Hospitals physicians (fte)	0.04	0.00	0.00	0.80
Hospital beds	0.04	0.00	0.00	0.73

*PIP* posterior inclusion probability, *SD* standard deviation, *Con.Pos.Sig.* posterior probability of a positive coefficient expected value conditional on inclusion, *GP* general practitioner, *fte* full-time equivalent

**Table 7:** Bayesian information criterion

Model specification	Total ACSC	Acute ACSC	Chronic ACSC
FE SEM (SWM_qc_)	− 2609.063	− 3213.829	− 2911.629
FE SEM (SWM_5nb_)	− 2608.017	− 3219.475	− 2905.941
FE SEM (SWM_dd_)	− 2596.923	− 3189.017	− 2904.421
FE SDEM (SWM_qc_)	− 2584.378	− 2573.898	− 2884.556
FE SDEM (SWM_5nb_)	− 2577.294	− 2584.288	− 2874.978
FE SDEM (SWM_dd_)	− 2563.670	− 2561.524	− 2872.024

*FE SEM* fixed effects spatial error model, *FE SDEM* fixed effect spatial durbin error model, *SWM* spatial weights matrix, *qc* queen contiguity, *5nb* 5 nearest neighbours, *dd* distance decay with 50 km cut-off

**Table 8:** Regression results for total, acute and chronic avoidable hospitalisations

	Total AH			Acute AH			Chronic AH		
	SWMqc	SWM_5nb_	SWM_dd_	SWM_qc_	SWM_5nb_	SWM_dd_	SWM_qc_	SWM_5nb_	SWM_dd_
University education	− 0.031	− 0.028	− 0.029	0.039	0.032	0.056	− 0.050	− 0.050	− 0.037
*Notstandshilfe* recipients	0.314	0.285	0.311**	− 0.238**	− 0.293***	− 0.183**	0.413***	0.399***	0.400***
Care allowance recipients	− 0.620***	− 0.621***	− 0.642***	− 0.189	− 0.169	− 0.242**	− 0.424***	− 0.425***	− 0.423***
Population density	− 0.000	− 0.000	− 0.000	− 0.000	− 0.000	0.000	− 0.000**	− 0.000	− 0.000
Population 15–64	0.705***	0.671***	0.786***	0.236***	0.120**	0.319***	0.432***	0.425***	0.425***
Population over 65	0.733***	0.670***	0.850***	0.183**	0.118	0.255***	0.415***	0.414***	0.438***
Female population	0.166	0.191	0.107	− 0.143	− 0.148	− 0.176	0.286	0.288	0.245
GPs per 1000 pop.	− 0.025**	− 0.023***	− 0.024***	0.012***	− 0.010***	− 0.012***	− 0.015***	− 0.015***	− 0.015***
Specialists per 1000 pop.	0.002**	0.003**	0.003**	0.001**	0.001	0.001**	0.001	0.001	0.001
GPs per specialists	0.012***	0.011***	0.011**	0.008***	0.006***	0.007***	0.004	0.004	0.004
Specialists over 70	− 0.062***	− 0.064***	− 0.062***	− 0.014	− 0.014	− 0.019	− 0.046***	− 0.048***	− 0.044***
Specialists 40–69	− 0.019**	− 0.019**	− 0.022**	− 0.006	− 0.006	− 0.009	− 0.017***	− 0.018***	− 0.019***
Hospital physicians	− 0.000	− 0.000	− 0.000	− 0.000	0.000	− 0.000	− 0.000	− 0.000	− 0.000
Spatial error term *ρ*	0.215***	0.240***	0.000	0.3131***	0.380***	0.094***	0.199***	0.147**	0.086***
BIC	− 2609.06	− 2608.02	− 2596.92	− 3213.83	− 3219.48	− 3189.02	−2911.63	−2905.94	− 2904.42

All models are estimated using a Spatial Error Model with regional and time fixed effects

*AH* avoidable hospitalisations, *GP* general practitioner, *SWM* spatial weights matrix, *qc* queen contiguity, *5nb* 5-nearest neighbours, *dd* distance decay 50 km cut-off, *fte* full-time equivalent, *BIC* bayesian information criterion

****p* < 0.01, ***p* < 0.05, **p* < 0.1

### Construction of the spatial weights matrices

When constructing a spatial weights matrix, one can broadly distinguish between binary and distance decay measures. The former identifies the number of regional links (e.g. *ω*_*i,t*_ = 1 if two regions are direct neighbours), whereas the latter incorporates the strength of regional links (e.g. inverse distance with *ω*_*i,t*_ = [*d*_*i,t*_] − 1, where d is the distance between centroids of *i* and *j*). For easier interpretation, it is common to normalize the spatial weights matrix so that for example the row sums equal 1. This so-called row-standardization leads the spatial lag operator, *W*, to produce a weighted average of the neighbouring observations. The coefficients, *θ*, *λ* and *ρ* measure endogenous, and exogenous spatial externalities as well as spatial dependence between the observation’s error terms, respectively.

## Footnotes

1 Between 5.7% (Latvia) and 12.4% (Switzerland) of the gross domestic product was spent on health in 2016 [1].

2 Primary healthcare usually refers to care provided at the first contact of the patient with the healthcare system, and is defined as the centre of a comprehensive, continuous and patient-centred prevention and care system. It is mostly delivered in the outpatient sector, i.e. the sector where patients are not formally admitted to a facility (e.g. hospital) [3]. In the remainder of this paper, the terms are used somewhat interchangeably, although the former rather refers to the treatment process, whereas latter refers to the location and organizational structure of the treatment.

3 Ambulatory care refers to health care not provided in hospitals but in outpatient physician offices.

4 Different studies include selected four digit ICD-10 codes from (a subset of) the following conditions: angina, asthma, cardiovascular diseases, cellulitis, chronic obstructive pulmonary disease, congestive heart failure, convulsions and epilepsy, dehydration and gastroenteritis, dental conditions, diabetes, nutritional, endocrine and metabolic, diseases of the blood, ear, nose and throat infections, gangrene, hypertension, influenza and pneumonia, iron deficiency anaemia, mental and behavioural disorders, nutritional deficiencies, pelvic inflammatory disease, perforated/bleeding ulcer, pyelonephritis and kidney/urinary tract infections, ulcers, osteoporosis, back pain.

5 International standard classification for education.

6 ‘Notstandshilfe’ is a form of financial social support that comes into force when period of unemployment exceeds the 1 year time frame for unemployment benefits.

7 Bias correction of the estimated error variance *σ*^2^ is necessary for large *N* and fixed *T* [36]. Even though the incidental parameter problem that occurs if panels are short leads to biased spatial fixed effects, the parameter *β* will be unbiased once *σ*^2^ has been corrected [34, 37].

8 Calculations are based on reimbursement data from 2013. On average an avoidable hospitalisation was associated with almost 2400 LKF points.
